# Editorial: Marbling fat in livestock

**DOI:** 10.3389/fvets.2022.1016428

**Published:** 2022-10-13

**Authors:** Min Du, Xiao Li

**Affiliations:** ^1^Department of Animal Sciences, Washington State University, Pullman, WA, United States; ^2^Key Laboratory of Animal Genetics, Breeding and Reproduction of Shaanxi Province, College of Animal Sciences and Technologies, Northwest A&F University, Yangling, China

**Keywords:** marbling, livestock, intramuscular adipocytes, lipid metabolism, meat

In animals used in food production, fat deposition is closely associated with production efficiency and meat quality. There are four major fat depots, including visceral, subcutaneous, intermuscular, and intramuscular fats, among which, only intramuscular fat (marbling fat) is critically important for the eating quality of meat. While most studies on intramuscular fat deposition concentrate on beef cattle traditionally, recent studies also emphasize its importance in determining the eating quality of pork, chicken, and other livestock species. Intramuscular fat development is due to both the formation of intramuscular adipocytes (hyperplasia) and their subsequent lipid accumulation (hypertrophy) (1). This special issue includes 12 articles on topics ranging from molecular mechanisms regulating intramuscular adipocyte formation and lipid accumulation. In addition, several species of livestock animals are covered.

Both intramuscular adipocyte hyperplasia and hypertrophy are affected by genetic, nutrition, and other environmental factors. In this issue, Sakowski et al. reviewed the genetic effects of European cattle breeds and their interaction with nutrition and other environmental factors. Due to their locations in the uterus, which restricts nutrient delivery to the fetuses, several piglets are born with much lower birth weights compared to their littermates.  Zhao et al. showed that low and normal birth weight piglets differed in their ability to accumulate intramuscular fat, which was not affected by glutamate supplementation.

The RNA world is becoming increasingly complicated due to developments such as coding mRNAs and non-coding RNAs. After the transcription of pre-mRNAs, they are processed through splicing to form mature mRNAs. Alternative splicing produces different mRNAs which are translated into different protein isoforms. In addition, the introns that are spliced out often contain microRNAs (miRNAs) that are increasingly recognized for their regulatory roles in cell differentiation and animal development. In addition, non-coding RNAs are complex, including long non-coding RNAs (lncRNAs), piwi-interacting RNAs (piRNAs), small nucleolar RNAs (snoRNAs), miRNAs, and others (2). While most non-coding RNAs are linear, recent studies also identified circular RNAs (circRNAs), which have higher stability and can regulate animal development *via* sponging miRNAs or through other mechanisms (3). Accumulating studies underscore the regulatory roles of non-coding RNAs in determining intramuscular fat development. In this issue, Li et al. profile the circular RNAs (circRNAs) in donkey muscle for their correlation with intramuscular fat deposition and found 127 differentially regulated circRNAs between low and high intramuscular fat groups, which target lipid metabolism. By comparing Wagyu and Chinese Red Steppe cattle, Yang et al. identified 115 differentially expressed and alternatively spliced genes, which affect lipid metabolism. Shu et al. found that several genes important for fatty acid metabolism have multiple alternative splicing, which was associated with intramuscular fat deposition in pigs. For miRNA, Xu et al. showed that miR-10a-5p affects intramuscular preadipocyte lipid accumulation by targeting Kruppel like factor 8 (KLF8) in goats.

Our understanding of the sources of intramuscular adipocytes remains limited. While most studies point to the fibro-adipogenic progenitor cells as the major source, the endothelial cells associated with blood vessels are an additional source (4). In addition, myogenic cells are a potential source of intramuscular adipocytes (5). Kim et al. showed that myoblasts are transdifferentiated into adipocytes in quails, which is promoted by the supplementation of retinoic acid. Following the formation of preadipocytes, these cells accumulate lipids to become mature adipocytes. Mao et al. showed that diacylglycerol acyltransferase 2 (DGAT2) stimulates lipid synthesis and thus adipocyte hypertrophy in pigeons. In addition, lipid accumulation differs among different fat depots. Through RNA-seq, Zhang et al. found that subcutaneous fat and visceral fat differ in the expression of genes regulating lipid metabolism. In addition, using RNA-seq, Bai et al. showed that long-chain acyl-CoA synthetase 1 (ACSL1) is correlated with unsaturated fatty acid synthesis in bovine adipocytes. Muscle fiber types and myosin heavy chain 3(MYH3) expression have been reported to affect marbling fat deposition. However, Huang et al. analyzed the variants in the MYH3 genes and found that there was no correlation between MYH3 variations and intramuscular fat content in several different pig breeds.

Finally, the marbling of beef carcasses is scored by inspecting the presence of intramuscular fat in the cross-section of *Ld* muscle. Depending on countries and geographical regions, the location of cross-cut is slightly different. In the US system, the marbling score is evaluated by ribbing between the 12^th^ to 13^th^ rib, while cuts at the fifth and tenth ribs are used in other countries. Liu et al. compared the marbling scores judged from the cut surface at the fifth and tenth ribs and, found that location did not affect the scores, showing ribbing location does not affect the marbling judgment.

In summary, articles in this issue cover several important aspects of intramuscular fat development and deposition, especially in RNA-seq and non-coding RNAs. While previous studies have focused on lipid metabolism and accumulation in mature adipocytes, new studies emphasize the importance of intramuscular fat formation, or the developmental sources of intramuscular adipocytes, in determining the degree of marbling fat deposition.

## Author contributions

MD and XL wrote the manuscript and agreed on the submitted version. All authors contributed to the article and approved the submitted version.

## Conflict of interest

The authors declare that the research was conducted in the absence of any commercial or financial relationships that could be construed as a potential conflict of interest.

## Publisher's note

All claims expressed in this article are solely those of the authors and do not necessarily represent those of their affiliated organizations, or those of the publisher, the editors and the reviewers. Any product that may be evaluated in this article, or claim that may be made by its manufacturer, is not guaranteed or endorsed by the publisher.
